# Force degradation of two orthodontic accessories analyzed in vivo and in vitro

**DOI:** 10.1186/s12903-023-03737-x

**Published:** 2023-12-14

**Authors:** Liu Yang, Chenxing Lv, Xiaomin Li, Jianying Feng

**Affiliations:** 1 Department of Stomatology, Hangzhou Traditional Chinese Medicine Hospital Affiliated to Zhejiang Chinese Medical University, Hangzhou, China; 2 Department of Orthodontics, State Key Laboratory of Oral and Maxillofacial Reconstruction and Regeneration, Key Laboratory of Oral Biomedicine Ministry of Education, Hubei Key Laboratory of Stomatology, School and Hospital of Stomatology, Wuhan University, Wuhan, China; 3 School and Hospital of Stomatology, Zhejiang Chinese Medical University, Hangzhou, China

**Keywords:** Elastomeric chains, NiTi coil springs, Force degradation, In vivo, In vitro

## Abstract

**Objective:**

To compare force degradation of elastomeric chains and NiTi coil springs in vivo and in vitro, and evaluate the effects of pre-stretched and reused elastomeric chains in the oral cavity during the time.

**Methods:**

In the in vitro groups, 4-unit elastomeric chains and NiTi coil springs with an initial force of 200 g were placed in dry air and artificial saliva. The volunteers wore clear retainers which were used to hold the sample of 4-unit chains, pre-stretched 4-unit chains, and NiTi coil springs with the initial force of 200 g in the in vivo groups. After the first 4 weeks, 4-unit specimens were stretched to 200 g again for another 4 weeks in vivo. The force value and the percentage of force degradation were recorded at each measurement time interval in the in vivo and in vitro groups.

**Results:**

The force degradation of elastomeric chains was greatest within the initial 4 hours, followed by a more stable phase after 1 week. The average force degradation of 4-unit elastomeric chains after 4 weeks was in vivo (64.8%) > artificial saliva (55.0%) > dry air (46.42%) (*P* < 0.05). The force degradation of NiTi coil springs in vivo (15.36%) or in artificial saliva (15.8%) was greater than in dry air (7.6%) (*P* < 0.05). NiTi coil springs presented a gentler force decay than elastomeric chains during the period (P < 0.05). In vivo, the force degradation of pre-stretched and reused elastomeric chains decreased less than the regular style(*P* < 0.05).

**Conclusion:**

The force degradation of the elastomeric chains and NiTi coil springs varied in different environments. NiTi coil springs presented a gentler force decay than elastomeric chains during the period. Orthodontists should consider the force degradation characteristics of orthodontic accessories in clinical practice.

## Introduction

Elastomeric chains were introduced to the dental profession in the 1960s [1]. Since then, they have been extensively used in orthodontics mainly for retracting canines and incisors after premolar extraction [2–5]. They were polymeric materials without ideal elasticity. One of the major characteristics of the elastomeric chains was that they absorbed saliva and experienced permanent deformation and force degradation in the oral environment due to the breakdown of internal bonds [6, 7].

Force degradation of elastomeric chains has been previously investigated in vitro studies [1, 3, 8–16]. Several studies have reported that the initial force of elastomeric chains slumped on the first day of use and kept this momentum. This variation range depends on several factors including the chain composition, environment, evaluation method, pre-stretched treatment, and initial force applied [8, 9].

Nickel titanium (NiTi) coil springs are also commonly used in space-closing systems. They are metallic materials with the unique property of super-elasticity. Compared to elastomeric chains, the force value of NiTi coil springs remained relatively constant during the period [14, 17, 18]. Since sliding mechanics have been widely applied to close residual extraction spaces, it is important to compare the force exerted by elastomeric chains and NiTi coil springs to assist orthodontists in selecting the appropriate devices.

In vitro studies simplified the system, allowing investigators to focus on a small number of factors and exclude some variables [15]. Despite the advantages of in vitro experiments, laboratory conditions cannot exactly simulate the real oral cavity. The oral environment of orthodontic patients seemed to be ideal for analyzing the characteristics of elastomeric chains and NiTi coil springs currently used under load. However, it was difficult to systematically assess the individual responses to the same orthodontic force and to standardize the distance or initial force. In vivo studies could better evaluate the factors affecting the force degradation of elastomeric chains and provide more reliable guidance for clinical applications of elastic force. Therefore, it is necessary to evaluate the characteristics of elastomeric chains and NiTi coil springs with the standardization both in vitro and in vivo.

The aim of this study was to evaluate the characteristics of force degradation of the two orthodontic accessories. This study tested the hypothesis that there would be a difference in force degradation between two orthodontic accessories and the force would decay differently in different environments.

## Materials and methods

Elastomeric chains (AlastiK transparent Spool Chain, 3 M Uniteck, USA) and NiTi coil springs (A-W-CS10506D, IMD, China) were used in the in vivo and in vitro experiments. Eighty pieces of 4-unit elastomeric chains and 60 NiTi coil springs were divided into 8 groups according to different environments and treatments, each with 20 samples (Table 1).

**Table 1:** Groups of two orthodontic accessories in different environments and treatments

Group	Experiment object	Environment	Pre-stretched treatment	Reused treatment
I	Elastomeric chains	Vivo	No	No
II	NiTi coil springs	Vivo	No	No
III	Elastomeric chains	Vivo	Yes^a^	No
IV	Elastomeric chains	Vivo	No	Yes^b^
V	Elastomeric chains	Air	No	No
VI	NiTi coil springs	Air	No	No
VII	Elastomeric chains	Artificial saliva	No	No
VIII	NiTi coil springs	Artificial saliva	No	No

^a^ Pre-stretched treatment: stretched twice the distance of the initial length of 4-unit elastomeric chains three times

^b^ Reused treatment: 4-unit elastomeric chains tested in Group I were reused in Group IV after 4 weeks

**Fig. 1:**
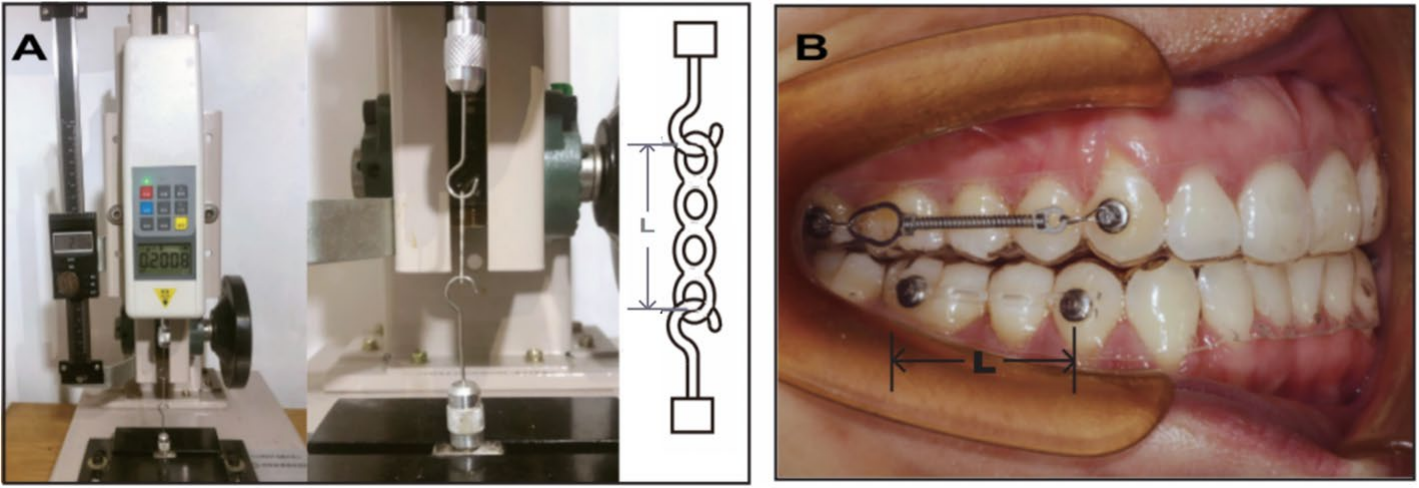
**A** Elastomeric chains tested by the testing machine. Detail of two hooks coupled to the testing machine, one was at its upper connection point and the other was fixed at the bottom. A diagram of the stretched elastomeric chains inserted on hooks. NiTi coil springs were tested using the same way. **B** Insertion of elastomeric chains and NiTi coil springs on buttons maintained by clear retains. L represented the distance of extension (mm)

The force values of the stretched samples were measured in a testing machine (load sensor 500G/5N, accuracy of 0.01 mm; Dongguan ZHIQU company, Dongguan, China) (Fig. 1A). Tensile readings were recorded in gram (g) force with a duration of 10 seconds for each sample. The length of the setting force (200 g) was measured for each group by electronic digital display Vernier (accuracy of 0.01 mm; Germany Master proof Company, Shanghai, China). Two hooks made of 1 mm stainless steel were used to hold each side of the specimens on the screws of the base (Fig. 1A). Then the average length of each group was the extension distance. All groups were extended to a specific distance using the same initial force of about 200 g and measured at intervals of initial, 4 hours, 1 day, 4 days, 1 week, 2 weeks, 3 weeks, and 4 weeks. These measurements were conducted by one investigator (L.Y) to ensure consistent handling of the test machine. With a pair of tweezers, each tested specimen was carefully removed from the retainer and transferred to the testing machine in the sequence of time intervals. In the process of stretching from a relaxed state to a specific distance, the force value of elastomeric chains, as shown on the test machine, gradually increased. When the specimen was stretched to a specific distance, the force value decreased rapidly at first, and then it tended to decline stably. The force value of the specimen was determined by taking the constant value of the test machine within 10 seconds, and each specimen was read three times for the average force value.

For the in vivo experiment, ten college students were chosen to wear clear retainers as volunteers from the School of Stomatology, Zhejiang Chinese Medical University. The inclusion and exclusion criteria for the volunteers were as follows: good physical and mental health, regular lifestyle, no history of orthodontic treatment, no bruxism, no dental crowding, and no moderate or severe periodontal disease. All participating subjects had signed an informed consent agreement. Silicon rubber impressions were collected to produce gypsum models of the maxillary and mandibular arches for each individual. A vacuum pressure film machine (Guang Ming Medical Device Company, Suzhou, China) was used to customize thermoplastic retainers of upper and lower arches. Light solidification glue was used to fix two buttons (diameter = 1 mm) on each quadrant of the clear retainers, representing the distance of Group I-IV extension to produce an initial force value (200 g). After the first 4 weeks, the elastomeric chains in Group I were removed from the clear retainers of different volunteers, and then the position of two buttons on the clear retainer was adjusted to represent the distance of reused elastomeric chains in Group IV. To prevent the elastomeric chains from breaking, volunteers had to wear clear retainers except for three basic meals all day, except during three main meals, and to avoid consuming any other beverages (Fig. 1B).

For the in vitro experiment, 4-unit elastomeric chains and NiTi coil springs were stretched to the setting force in retainers, as previously done. Instead of being worn in volunteers’ mouths, retainers were placed in a dry environment with a constant temperature of 25 °C and relative humidity of 60%. They were also immersed in artificial saliva with a constant temperature of 37 °C and a pH of 6.7. The artificial saliva, which closely resembles natural saliva, consists of the following components: KCl (0.4 g/L), NaCl (0.4 g/L), CaCl_2_·2H_2_O (0.906 g/L), NaH_2_PO_4_·2H_2_O (0.690 g/L), Na_2_S·9H_2_O (0.005 g/L), and Urea (1 g/L). The solution was titrated to a pH of 6.7 using NaOH (5 mol/L) and replaced once a week. Force measurements were made at the same time intervals as the in vivo experiments.

### Statistical analysis

The collected data was analyzed by the SPSS software (Version 22.0, IBM, Armonk, NY). The means and standard deviations of force degradation were calculated for each group at each time interval. One-way ANOVA test with Bonferroni correction and t-test were used to identify statistically significant intragroup and intergroup differences at a 5% significance level.

## Result

**Fig. 2:**
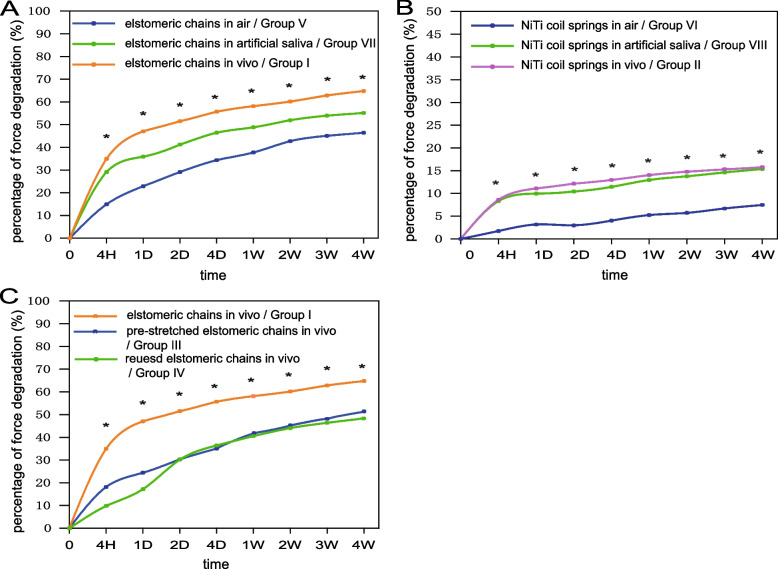
Force degradation (%) over time of elastomeric chains and NiTi coil springs groups. **A** * Denotes statistically significant differences among three groups at the same time point. **B** * Denotes statistically significant differences between group VI and group II or group VIII at the same time point. **C** * Denotes statistically significant differences between group I and group III or group IV at the same time point

The means and standard deviation of the force value and the force degradation percentage of all groups at each measured time interval were shown in Table 2. The curve extension of elastomeric chains in different environments showed a decreasing trend of force value over time. Within the initial 4 hours, the degradation of the elastic force was the greatest, the average degradation rate of the elastic force was as follows: in vivo (35.9%) > artificial saliva (29.1%) > dry air condition (14.9%) (*P* < 0.05). After that, the degradation of force value decreased steadily. After 4 weeks, the average degradation of the 4-unit elastomeric chains force were as follows: in vivo (64.8%) > artificial saliva (55.0%) > dry air condition (46.4%) (*P* < 0.05) (Fig. 2A). At the same time point, the force degradation of NiTi coil springs decreased more obviously in vivo and in artificial saliva compared with the dry air condition (P < 0.05). However, there were no statistically significant differences between the in vivo and artificial saliva groups. After 4 weeks, the force degradation of NiTi coil springs was 15.2% in vivo, 15.1% in artificial saliva, and 7.6% in the dry air (Fig. 2B).

**Table 2:** Means and standard deviations of the extension force (g) and percentage of force degradation (%) for each group

group	force	initial	4 hours	24 hours	2 days	4 days	1 weeks	2 weeks	3 weeks	4 weeks
I	g	201.2 ± 6.1	129.6 ± 6.5	107.1 ± 6.0	98.0 ± 4.5	89.6 ± 5.5	84.6 ± 4.3	80.55 ± 5.0	75.2 ± 4.7	71.2 ± 4.1
	%	0 ± 0	35.9 ± 2.8	47.1 ± 1.8	51.5 ± 1.5	55.7 ± 3.0	58.1 ± 2.5	62.8 ± 2.4	62.8 ± 2.5	64.8 ± 2.2
II	g	198.8 ± 2.2	182.2 ± 3.6	179.0 ± 4.0^a^	178.0 ± 5.7	176.0 ± 6.6	173.0 ± 7.5^a^	171.4 ± 8.6^a^	169.7 ± 8.3^a^	168.2 ± 6.5
	%	0 ± 0	8.3 ± 1.4	10.0 ± 1.6	10.4 ± 2.7	11.4 ± 3.3	13.0 ± 3.7	13.8 ± 4.3	14.6 ± 4.3	15.4 ± 3.6
III	g	203.9 ± 7.0	166.9 ± 5.3	154.1 ± 5.0	142.1 ± 5.9	132.5 ± 5.6	119.8 ± 4.7	110.66 ± 4.8	105.9 ± 5.8	99.8 ± 6.8
	%	0 ± 0	18.1 ± 3.1	24.4 ± 3.5	30.3 ± 3.8	35.0 ± 3.3	41.2 ± 2.8	45.7 ± 2.8	48.0 ± 3.6	51.1 ± 3.4
IV	g	201.5 ± 4.4	181.7 ± 5.5	168.8 ± 4.6	138.7 ± 4.0	128.0 ± 4.6	119.6 ± 5.6	112.51 ± 6.0	107.9 ± 5.8	103.9 ± 6.0
	%	0 ± 0	9.8 ± 3.2	17.2 ± 4.0	30.2 ± 3.5	36.5 ± 2.6	40.6 ± 3.0	44.1 ± 3.2	46.5 ± 3.2	48.4 ± 3.2
V	g	201.9 ± 5.2	171.8 ± 6.3	155.7 ± 7.1	143.1 ± 5.6	132.5 ± 5.9	125.7 ± 4.5	115.7 ± 6.0	110.9 ± 4.6	108.2 ± 5.0
	%	0 ± 0	14.9 ± 3.1	22.9 ± 2.9	29.2 ± 2.3	34.4 ± 2.9	37.7 ± 1.5	42.8 ± 2.1	45.0 ± 2.2	46.4 ± 2.3
VI	g	200.3 ± 2.5^a^	196.8 ± 3.0^a^	193.9 ± 2.7^a^	194.3 ± 2.8^a^	192.2 ± 2.9^a^	189.7 ± 3.1	188.8 ± 2.9^a^	186.8 ± 2.7^a^	185.3 ± 2.5
	%	0 ± 0	1.7 ± 0.6	3.2 ± 0.9	3.0 ± 1.2	4.0 ± 1.1	5.2 ± 1.6	5.7 ± 1.4	6.7 ± 1.7	7.5 ± 1.5
VII	g	203.0 ± 4.7	143.9 ± 5.9	130.1 ± 5.1	119.3 ± 5.0	108.7 ± 6.7	103.9 ± 7.0	97.54 ± 4.3	93.5 ± 4.5	91.0 ± 3.4
	%	0 ± 0	29.1 ± 3.0	35.8 ± 3.1	40.9 ± 3.0	46.1 ± 3.9	48.5 ± 4.0	51.8 ± 2.3	53.6 ± 2.7	55.0 ± 2.1
VIII	g	200.5 ± 1.5	183.2 ± 3.6^a^	178.2 ± 3.7^a^	176.1 ± 4.6^a^	174.5 ± 3.4^a^	172.4 ± 3.3^a^	170.8 ± 3.8^a^	169.8 ± 3.1^a^	168.8 ± 4.4
	%	0 ± 0	8.6 ± 1.5	11.1 ± 1.8	12.1 ± 2.2	13.0 ± 1.8	14.0 ± 2.0	14.8 ± 2.2	15.3 ± 1.9	15.75 ± 2.5

^a^Denotes no statistically significant differences in the force value between the extension marked and the following extension in the same group (*P*>0.05)

For the in vivo groups, the force degradation of elastomeric chains and NiTi coil springs were compared. Under the same force value (200 g), elastomeric chains showed a significantly higher degradation rate than NiTi coil springs at all time intervals (*P* < 0.05). After 4 weeks, the degradation of elastomeric chains was about 4 times higher than that of NiTi coil springs.

Degradation of the elastic force was compared under the same situation but with different treatments. In vivo, pre-stretched 4-unit elastomeric chains (Group III) and reused 4-unit elastomeric chains (Group IV) showed less force degradation than untreated elastomeric chains (Group I) at all time intervals (*P* < 0.05). After 4 weeks, the force degradation rate of Group III was 51.1% and Group IV was 48.4%, both of which were less than that of Group I (64.8%) (Fig. 2C).

## Discussion

This study was conducted to test the hypothesis that there would be a difference in force degradation between two accessories and among different environments. The initial force of all groups was standardized at 200 g in this study, as it lay within the optimal force magnitude for space closure and could be considered equal for evaluating the force degradation. Two orthodontic accessories were fixed between the button points correspondingly in the personalized retainer to prevent direct force application to the teeth. In this way, the actual dynamic situation of orthodontic treatment could be simulated, especially for space closure.

The present study found that the force degradation of elastomeric chains in vivo (64.8%) was greater than in dry air (46.4%) and in artificial saliva (55.0%) after 4 weeks, which seemed to be a similar trend to those from previous in vitro studies [3, 10–12, 15, 16, 19]. The percentage of force degradation varies among studies because of differences in the composition and size of elastomeric chains. Furthermore, consistent with findings from previous studies [10, 11, 15, 16, 19, 20], elastic force experienced the most significant degradation within the initial 4 hours, followed by a stable phase after 7 days. A recent report [21], using the same method to evaluate the properties of latex chains in vivo and in vitro, found that the force value of latex chains decreased faster in vivo than in vitro and exhibited a high percentage of force loss at the early stages. The latex chains and elastomeric chains were found to exhibit similar patterns of force degradation. One possible explanation for this similarity is that both materials are made of polyurethane, which lacks ideal elasticity and is sensitive to prolonged exposure to intraoral conditions. Considering the degradation regulations of elastomeric products, a slightly higher initial force value might be appropriate for clinical applications [4, 22, 23].

In this study, the force degradation of NiTi coil springs in dry air (7.6%) is relatively less than in vivo (15.2%) or artificial saliva (15.0%), which was similar to previous studies [17, 18, 24]. The reason was that the NiTi coil spring with shape memory could be less influenced by the humidity and pH of the environment [25]. Notably, compared with elastomeric chains, NiTi coil springs were more resistant to force degradation and presented a gentler and more progressive force degradation. After 4 weeks, the elastomeric chains showed a significantly greater force decay (64.8%) than that presented by the NiTi coil springs (15.2%), corroborating the results of other investigations [26]. Compared to elastomeric chains, NiTi materials with super-elasticity are supposed to overcome the issue of rapid force decay and provide light and continuous forces over a wide range of activation [24, 25]. However, they could easily accumulate food particles, which were difficult to remove and might be detrimental to oral hygiene. Thus, elastomeric chains remain popular in clinical practice due to their affordability, practicality, and ease of cleaning. Additionally, in space closure systems, elastomeric chains were considered to deliver intermittent forces that can provide time for alveolar bone remodeling and reduce side effects such as root resorption [23]. It is imperative for clinicians to be aware of the characteristics of the force delivery system to facilitate optimal selection.

To counteract the rapid loss of force of elastomeric chains, pre-stretching the elastomeric chain has been suggested to reduce force decay before it is stretched with tension [3, 22]. After the pre-stretching treatment, the elastomeric chains experience deformation, leading to an increased length after the post-stretching process. In clinical practice, for instance, elastomeric chains that initially required 5 units are now relieved by only 4 units to generate the same initial force value. The present study chose a method of pre-stretching treatment that was applicable at the chairside and compared its effect on the behavior of elastomeric chains with the same unit and initial force value. The extension distance of the pre-stretched group differs from that of the non-pre-stretched group to achieve the same initial force. This contrasts with some studies that used a uniform stretching distance like 30 mm [3], which could lead to different initial forces. It was found that with the same initial force (200 g), the force degradation of the pre-stretched groups was smaller than that of the non-pre-stretched groups after 4 weeks. These findings were in agreement with the results of Chang et al. [27], who also found that pre-stretching treatment was not accompanied by disadvantages such as permanent deformation. Whether the pre-stretching treatment of elastomeric chains with different units or different initial force values will affect the force degradation of elastomeric chains needs further study.

Since there was a loss of force over time, clinicians conventionally removed the original chains and replaced them with new chains in a patient’s mouth every 4 to 6 weeks. It is unknown whether the original chains that were reused and stretched again have the same mechanical performance as the non-reused ones [28]. In Group IV, 4-unit elastomeric chains tested in Group I after 4 weeks were reused in vivo**.** In the same situation, the results showed that the force degradation of Group IV was less than non-reused elastomeric chains at all time intervals (*P* < 0.05). In this experiment, volunteers were asked to wear the retainers except during meals. This approach helped to eliminate various factors that could potentially affect the results, such as the patient’s diet, masticatory force, tooth-brushing, the presence of bacterial enzymes, and temperature changes. All the above factors could lead to permanent deformation and increase the force degradation of elastomeric chains. Hence, the treatment of reused elastomeric chains should be chosen based on the actual situation.

This study had several limitations. The extension distance of orthodontic accessories might decrease during the process of tooth space closure, whereas the extension distance remained constant in our study. Consequently, the force degradation of two orthodontic accessories might be influenced by changes in distance. Although volunteers were required to wear clear retainers for about 22 hours a day, the wear time of the elastomeric chains was less than that of the actual clinical situation (24 hours a day). Therefore, the in vivo group was unable to fully replicate the actual situation of orthodontic therapy. Considering that, the force degradation of elastomeric chains in clinical practice might be greater than the results of our in vivo study.

## Conclusion

1. The force degradation of the elastomeric chains and NiTi coil springs varied in different environments. NiTi coil springs presented a gentler force decay than elastomeric chains during the period.
2. Using pre-stretched and reused elastomeric chains might decrease the rate of force degradation.
3. In clinical practice, orthodontists should consider the force degradation characteristics of two orthodontic accessories when using them for space closure.

## Data Availability

All data generated or analyzed during this study are included in this published article.
